# The Influence of Wheel/Rail Contact Conditions on the Microstructure and Hardness of Railway Wheels

**DOI:** 10.1155/2014/209752

**Published:** 2014-01-16

**Authors:** Paul Molyneux-Berry, Claire Davis, Adam Bevan

**Affiliations:** ^1^Institute of Railway Research, School of Computing and Engineering, University of Huddersfield, Queensgate, Huddersfield HD1 3DH, UK; ^2^School of Metallurgy and Materials, University of Birmingham, Edgbaston, Birmingham B15 2TT, UK

## Abstract

The susceptibility of railway wheels to wear and rolling contact fatigue damage is influenced by the properties of the wheel material. These are influenced by the steel composition, wheel manufacturing process, and thermal and mechanical loading during operation. The in-service properties therefore vary with depth below the surface and with position across the wheel tread. This paper discusses the stress history at the wheel/rail contact (derived from dynamic simulations) and observed variations in hardness and microstructure. It is shown that the hardness of an “in-service” wheel rim varies significantly, with three distinct effects. The underlying hardness trend with depth can be related to microstructural changes during manufacturing (proeutectoid ferrite fraction and pearlite lamellae spacing). The near-surface layer exhibits plastic flow and microstructural shear, especially in regions which experience high tangential forces when curving, with consequentially higher hardness values. Between 1 mm and 7 mm depth, the wheel/rail contacts cause stresses exceeding the material yield stress, leading to work hardening, without a macroscopic change in microstructure. These changes in material properties through the depth of the wheel rim would tend to increase the likelihood of crack initiation on wheels toward the end of their life. This correlates with observations from several train fleets.

## 1. Background

### 1.1. Objective

This paper aims to investigate the influence of the as-manufactured steel microstructure and hardness along with the influence of wheel/rail contact conditions on the observed damage (such as rolling contact fatigue (RCF) cracks and plastic flow) on the tread of railway wheels.

Previous studies of wheel wear and RCF crack initiation and propagation have used several approaches:

1. analytical or finite element modelling of simplified conditions: such simplifications may include simplified geometry, elastic and/or isotropic material, or one repeated loading condition [1–6],
2. twin-disc tests and material analysis, which can be well controlled but may not be realistic in scale, contact geometry, loading conditions, material properties, or cycles to failure [7–9],
3. dynamic simulations of wheel/rail contact conditions and stress fields in the wheel, which can consider a wide range of conditions and influences but require some simplifications [10–12],
4. empirical or statistical models based on observations of wheel damage in service, which can be useful but may not be widely applicable and are unable to account for some observations [13–17],
5. material analysis of new and used wheels, which can help to understand the variation in material properties but where the stress history of the wheel may not be well defined [18–21].

All these areas of research are very valuable, but the current “state of the art” has not achieved an integrated deterministic model of wheel damage owing to the complexity of the conditions [22, 23]. The authors of this paper have combined the findings of simulations, wheel damage observations and material analysis ((3), (4), and (5) in the previous list) to provide a dataset which can be used to refine empirical models or identify the ways in which finite element models could be enhanced, as a step towards an integrated deterministic model. Given the relatively broad scope of the investigations and the space available, it is not practical to provide a fully detailed description of each analysis summarised in the paper.

### 1.2. Railway Wheel Observations

Railway wheels operate in a demanding environment with high normal contact forces and significant tangential forces. The resulting stresses often exceed the yield stress of the as-manufactured wheel material, leading to plastic flow, wear, and fatigue damage.

Previous work on disc-braked passenger rolling stock has shown that different categories of observed surface damage occur at various positions across the wheel profile. These categories of damage can be related to wheel/rail contact conditions and forces derived from dynamic simulations of vehicle operating conditions [10]. However, there can be large variations in the rate at which observed wear and fatigue damage accumulates.

Wheel damage observations from a UK regional diesel multiple unit (DMU) fleet form the core of this work: the vehicles have an axle load of approximately 15 tonnes and operate over routes including prolonged steep gradients and sharp curves. The wear and surface appearance of wheel damage on this fleet has been examined in detail on nine vehicles over the lifetime of the wheels, including observations of sub-surface damage during reprofiling. This has been backed by analysis of wheel reprofiling trends for the entire fleet of over 100 vehicles over two wheel lifetimes.

The wheels exhibit a range of wear rates and a variety of crack damage types on motored and trailer wheelsets, which have been related to the operating conditions in a previous paper [10]. RCF is the dominant wheel damage mechanism, and the wheels are now usually turned on a preventive distance-interval basis (approximately 140,000 miles *≈* 224,000 km) to control the propagation depth of RCF cracks, thereby limiting the material removed on the lathe. In a recent survey of wheel damage on over 90% of UK passenger fleets, RCF was a common damage mechanism and the damage patterns on the monitored regional DMUs are representative of the majority [24].

**Figure 1:**
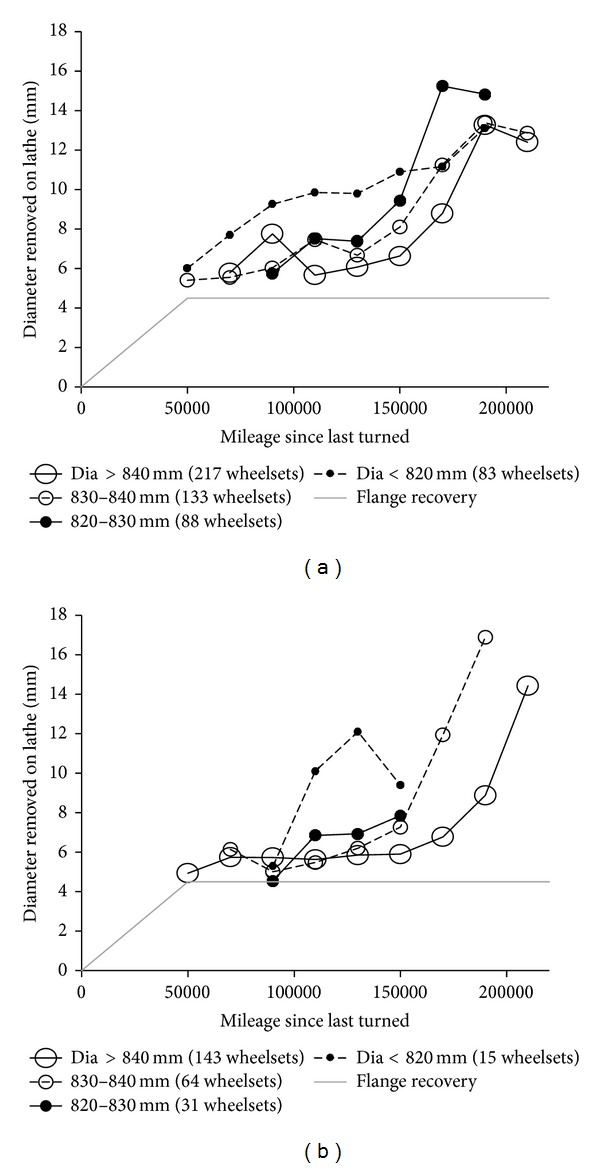
Influence of wheel diameter on average depth of material removal at reprofiling, as a function of mileage since last turned. This example is for the leading wheelset 1, which is most commonly affected by RCF. Wheels turned because of other damage types (e.g., flats) or for parity are excluded. (a) Wheels from all manufacturers. (b) Wheels from only one manufacturer. It is usually necessary to remove approximately *φ*5 mm to recover the flange thickness; deeper cuts indicate that RCF damage has propagated more deeply. Both graphs show a trend that RCF becomes serious more quickly on wheels of smaller diameter.

On the regional DMU fleet, RCF damage is observed to form much more rapidly on wheels which are reaching the end of their life, as shown in Figure 1. Most of the surveyed fleets [24] suffering from RCF also reported the same observation. The authors suggest a number of possible reasons for this effect, including the following:

(i)  change in material properties (hardness and microstructure) with depth below the as-manufactured tread surface: this is investigated in this paper and believed to be significant;
(ii) reduction in residual compressive stress remaining from the manufacturing process (the high beneficial residual compressive hoop stresses at the surface are lost due to plastic flow, wear, and material removal at reprofiling): variations in residual stress have been measured [25, 26] and some models have considered their influence on RCF damage [23], which may be significant. The authors are conducting further experiments to determine the changes in residual stress through the life of a wheel;
(iii) increased contact stress (due to smaller wheel radius): the change in wheel diameter is *≈*6% which would increase the Hertzian contact stress by *≈*2%. While this may be a contributory factor, it is probably not sufficient to be the sole cause;
(iv) higher number of stress cycles for a given wheelset mileage (due to increase in number of wheel revolutions): the change in revolutions/mile is *≈*6%. While this may be a contributory factor it is probably not sufficient to be the sole cause;
(v) reduced wheelset rolling inertia increasing the probability of wheel spin or slide events: on the monitored fleet, improvements in the wheel slide protection and traction control software have reduced the occurrence of these events, and they are excluded from the data presented in Figure 1. However, they may be a contributory factor on some fleets;
(vi) “human factors” issues: in an attempt to prolong wheel life, the lathe operator may minimise the cut depth on a small wheel and thereby not remove all previous damage: this has been identified as a problem on some fleets and may be relevant but is difficult to assess and quantify. Considering the experience of the staff at the depot and the detailed monitoring undertaken by the authors, this is unlikely to have a significant influence on the monitored fleet. 

### 1.3. Wheel Manufacturing Process

Modern multiple-unit trains running in the UK are fitted with one-piece forged wheels, usually to the rim-chilled hypoeutectoid grade known as R8T or ER8T. The chemical composition and properties of the steel are tightly defined by the relevant national and international standards [27, 28].

The manufacturing process for these wheels is complex [29] and also well described in online literature [30, 31]. After rough machining, the wheel is heated to approximately 900°C (austenitisation) and the wheel rim (only) is then rapidly water-quenched to 300°C. The wheel is then left to slowly air-cool. This process achieves two key goals.

- The rapid rim quenching makes the wheel rim material harder than the wheel centre and thus more resistant to wear and crack initiation. Meanwhile the wheel web and centre are softer and therefore less prone to crack propagation.
- The slow contraction of the web and centre during air-cooling causes compressive residual stresses in the already-transformed wheel rim, thereby making it more resistant to crack propagation. Meanwhile the wheel web and the inner part of the rim carry residual tensile stresses.

Following cooling, the wheel is then tempered at approximately 500°C. These processes are key to achieving a wheel which is resistant to surface damage.

### 1.4. Hardness Measurements of New Wheels

There are a number of papers describing measurements of material properties in wheels; these have considered a range of wheel steels. Tests on new wheels of differing materials are detailed in [18, 32]; these include results for SAE1060 and R7 steels which are similar to R8T. These papers describe the results of a range of material property tests, at different locations within the wheel rim. They demonstrate variations in hardness, microstructure, and fatigue behaviour through the depth of the wheel rim.

**Figure 2:**
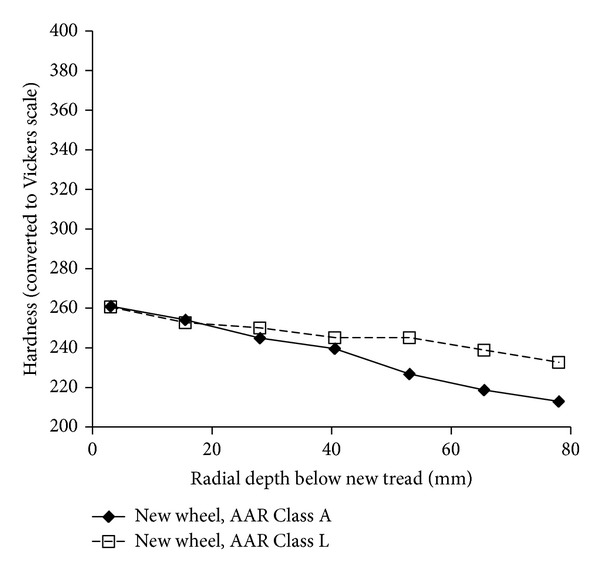
Trends of hardness with depth below the tread surface for new quenched wheels, adapted from Grundy [9].

In the USA, an extensive wheel material testing programme was carried out [19]. Rockwell hardness values across the entire wheel rim cross section are presented for new AAR Class A and Class L wheels. Both of these are rim-quenched; wheel steel grades in the USA are defined differently from those in Europe, but R8T material is broadly similar to Class A. These results are summarised in Figure 2; hardness values have been averaged across the wheel tread (excluding the flange area) and have been converted to Vickers hardness for comparison with other results presented later.

The results in Figure 2 for new wheels show how the hardness reduces quite linearly with depth as a consequence of the manufacturing process. It is expected that a new R8T wheel will show a similar trend, although with slightly higher hardness throughout.

## 2. Simulation of Contact Conditions

Dynamic simulations have been carried out to predict the wheel/rail contact conditions and forces for the regional DMU fleet currently being monitored by the authors. The simulations represented operation on the 68 km route where these trains run most frequently [10]. Vampire rail vehicle dynamics software has been used; the transient analysis is a time-stepping integration method which has been thoroughly validated [33] and is the industry standard in the UK. The inputs were made as detailed as possible using currently available data to accurately represent the duty of the fleet and are listed in Table 1.

Outputs from the simulations include the position and shape of the contact patch and the normal and tangential forces. These outputs were also postprocessed for input to the CONTACT software [34]. This software includes a more detailed contact model using non-Hertzian theory and was used to estimate the stress field underlying each contact.

**Figure 3:**
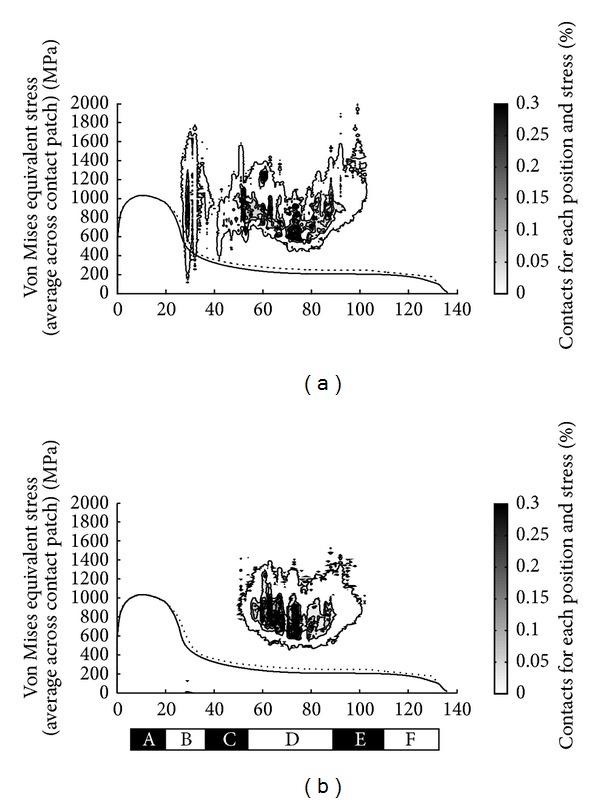
Distribution of contact conditions across a wheel profile for leading (a) and trailing (b) axle. The y axis shows the Von Mises equivalent stress (based on the average stress for each contact patch). Contours indicate the prevalence of contacts under each condition, with darker shades of grey representing a higher percentage of contacts, such as the frequent contacts in the centre of the tread at low stress. Higher stresses occur in other locations across the wheel profile when leading, but these are less frequent. On the trailing wheelset, the stress cycles are mostly in the running band (Region D) and with moderate stress levels. Approximately half the cycles on a given wheelset are experienced when it is leading the bogie, and half when it is the trailing wheelset.

Figure 3 shows the predicted distribution of contacts across the wheel tread of a powered wheel, based on Vampire simulations with the associated Hertzian contact assumptions. Figure 3 also shows the new and fully worn wheel profiles and identifies six regions (A to F) across the wheel profile which are described below.

The number of stress cycles in each region, occurring since the wheelset was last turned on the lathe, can be estimated from the simulation results and extrapolated for the total distance since turning. These are shown in Table 2 for the conditions shown in Figure 3.

The field side of the wheel usually only contacts the rails at turnouts, in particular at worn crossings. These have not been included in the simulations, so no stress cycles are predicted in this region in Figure 3. In practice, there are occasional contacts here, and Table 2 shows an estimated value based on the number of crossings traversed, the proportion of these that are worn, and the area of the field side band influenced.

**Figure 4:**
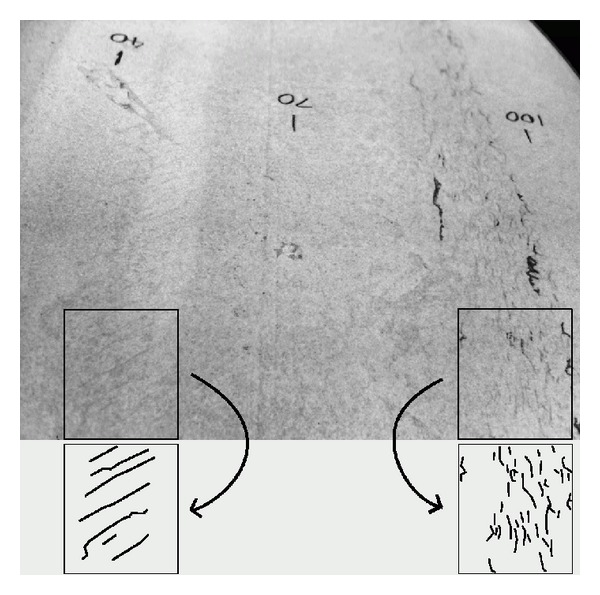
Two bands of cracks on surface of wheel, highlighted with dye penetrant. The flange is to the left and the field side to the right. Inverted numerals (40, 70, and 100) indicate distance from the back of the flange in millimetres. A manually enhanced sample of each crack band is shown below the original in the boxes. Flange root (Region C, left) has long angled surface cracks. Field side band (Region E, right) has circumferential cracks; some of these are longer and penetrate more deeply.

**Figure 5:**
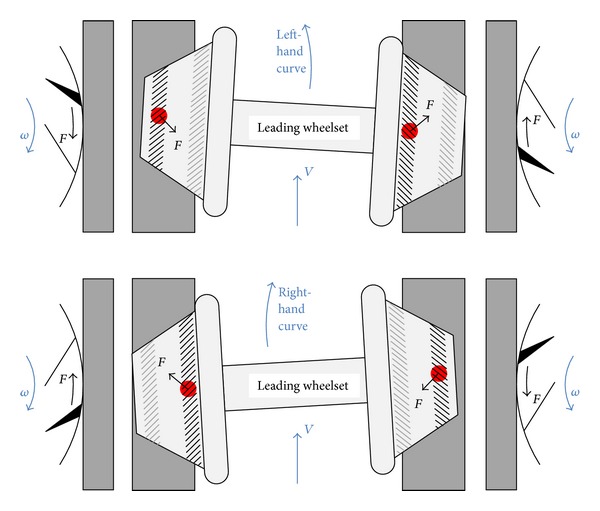
Orientation of leading wheelset on left and right hand curves, showing position of wheel/rail contact, direction of tangential force, and formation of two bands of cracks. The cracks near the flange of the wheel tend to be forced closed before contacting the rail and do not tend to propagate deep into the wheel. In contrast, the cracks near the field side of the wheel are forced open before they contact the rail; fluid entrapment and pressurisation in the contact can propagate these cracks into the underlying material.

The simulated contact conditions can be associated with observed damage on the various regions across the wheel profile and with the changes in hardness and microstructure. The conditions in these regions are summarised in the following paragraphs and are described in more detail in [10]. The two observed crack bands are shown in Figure 4, while Figure 5 shows the curving conditions where this damage is formed on the leading wheelset of a bogie.

### 2.1. Region A: Flange Tip

This part of the wheel would not normally contact the rails, and no contacts are predicted in the simulation. The wheel sample does not show any evidence of contact or wear in this region.

### 2.2. Region B: Flange Face

The leading wheelset flange contacts the outer rail on sharp curves. The stresses can be high, especially the tangential stress if the contact is not lubricated. Observed surface damage is primarily wear.

### 2.3. Region C: Flange Root

**Figure 6:**
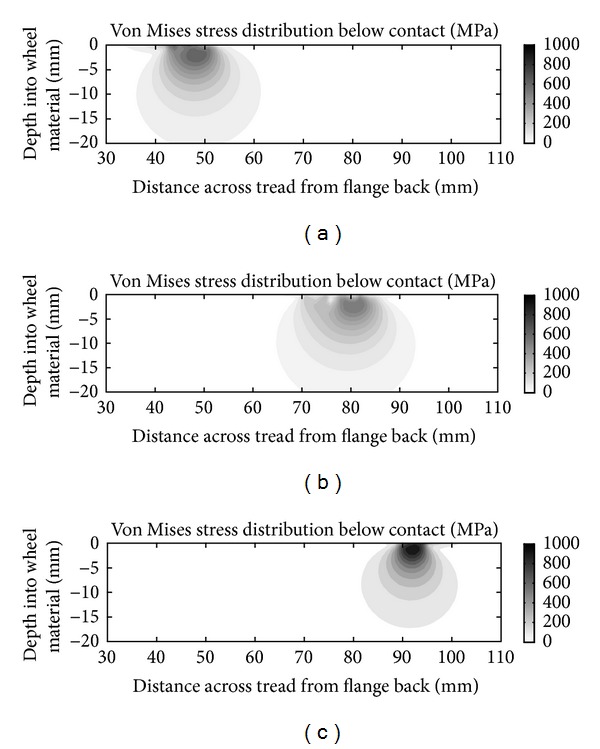
(a) Typical subsurface stress field in the flange root, Region C. (b) Typical subsurface stress field in the running band, Region D. (c) Typical subsurface stress field in the RCF band, Region E.

This portion of the leading wheel contacts the outer rail on moderate curves; there can be 2-point contact in regions B and C on sharp curves. This can cause significant normal and tangential stresses as the flange root is usually unlubricated. A typical sub-surface stress field is shown in Figure 6(a). Observed surface damage includes fine surface cracks; these do not tend to propagate deeper, most likely because the direction of the tangential forces does not promote propagation by fluid entrapment [10]; crack growth may also be counteracted by higher wear rates in this region.

### 2.4. Region D: Running Band (Centre of the Wheel Tread)

**Figure 7:**
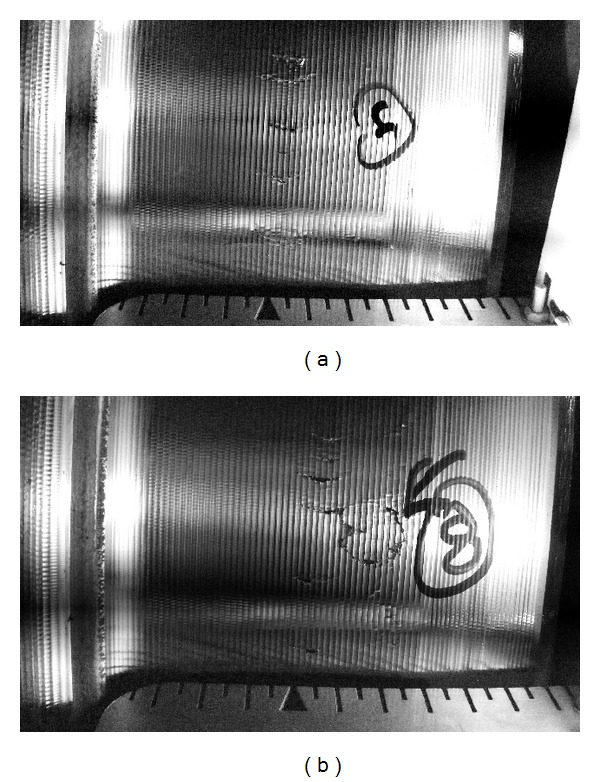
Subsurface cracks in centre of wheel tread, observed during turning of wheel on lathe: 2 images from different parts of the same wheel. Cracks were not visible on surface, although there was an unusual 0.4 mm out of roundness identified by the lathe in this region. Numerous curved transverse cracks appeared after 1.5 mm of material was removed from the surface; some appeared circular. Flange to left-hand side, tread datum 70 mm from flange back, is indicated by the arrow shape at the bottom of the image, with scale in 5 mm increments.

This portion of both leading and trailing wheels is used when running on straight track, which is the most common running condition. The trailing wheelset often contacts in this region on curves as well. There are many cycles with moderate normal stresses. The relatively small tangential forces associated with traction or braking mean that the peak stress tends to occur below the surface, as shown in Figure 6(b). Unlike the other regions of the wheel, the running band experiences a similar number of stress cycles in each rolling direction. On the observed (disc-braked) fleet, surface damage in this region is limited to mild wear; sub-surface transverse cracks have been observed on individual wheels; these may be influenced by material quality. Examples of this type of crack are shown in Figure 7.

### 2.5. Region E: RCF Band

This part of the leading wheel contacts the inner rail on curves; the opposite wheel is in flange face or flange root contact. There are fewer contacts in these conditions, but they typically occur with higher stresses and correspond with an increase in material hardening and observed RCF cracking. Contacts in this band can also occur on the trailing wheelset when curving at high cant deficiency, but these are less frequent and generally at lower stress. On many vehicles, cracks in this region are angled (as shown in Figure 5), but on the powered axles of the monitored fleet they are close to circumferential owing to the influence of traction forces. The surface direction of the observed cracks is approximately normal to the predicted tangential forces [10]. A typical sub-surface stress field is shown in Figure 6(c).

### 2.6. Region F: Field Side

This part of the wheel usually only contacts the rails at worn crossings in turnouts. In these conditions, the wheel can experience high normal stresses owing to the rapid transfer of vertical load on a nonconformal profile and high tangential stresses owing to the large rolling radius difference when contacting in this location. However, the number of stress cycles in this region is very small.

## 3. Wheel Hardness Measurements

Five used wheels from UK multiple-unit trains have been sectioned by the authors and their hardness and microstructure examined. All these wheels are of the R8T steel grade, but represent three different wheel manufacturers, three train types (with different wheel designs), and various stages during the life of the wheel. All had some degree of RCF damage in the field side band (Region E) which is typical of these fleets [24], and two also had some shallower RCF cracks in the flange root band (Region C). Two or more sections from each wheel were examined.

The results presented here focus on an end-of-life wheel from the regional DMU fleet being monitored by the authors. However, all the wheels sectioned by the authors showed similar trends in hardness and microstructural shear, and selected results from these are included. Given the similar trends in observed RCF damage on many UK fleets [24], the findings should have wider relevance.

The selected wheel was from a powered axle, with a stress history consistent with that presented in Figure 3 and Table 2. The wheel had run for 4.5 years and 800,000 miles (*≈*1,300,000 km), being reprofiled four times, and had been renewed on reaching the minimum permissible diameter. It had run nearly 170,000 miles (*≈*270,000 km) since last being turned on the wheel lathe. Some circumferential RCF cracking was apparent towards the field side of the tread, and a few fine surface cracks were just visible in the flange root area (as shown in Figure 4).

**Figure 8:**
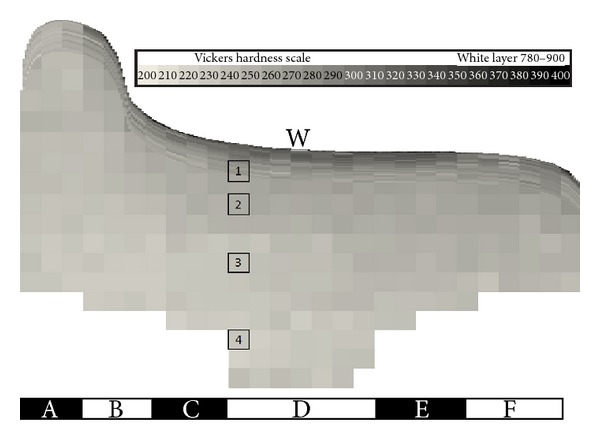
Hardness map for end-of-life regional DMU wheel. The numbers indicate locations of microstructure images shown in Figure 19.

The wheel was sectioned and a hardness map of the rim cross section was made. Macrohardness tests with a 30 kgf load were used to map the hardness at 5 mm increments across the entire section. Microhardness tests with a 1 kgf load were also carried out to achieve a higher resolution in the near-surface regions to a depth of 7.5 mm below the surface. In both cases a Vickers pyramidal indenter was used; the results are presented on the Vickers hardness scale (200 HV to 400 HV) in Figure 8.

Vickers hardness measurements do have an inherent variability owing to the inhomogeneity of the steel and measurement accuracy of the indentation. However, the measurements do show clear trends without excessive variability. The macro- and microhardness measurements were made on adjacent slices of the wheel rim, and in the regions of overlap there was a good match between the values obtained by the two methods. This gives confidence in the results obtained.

**Figure 9:**
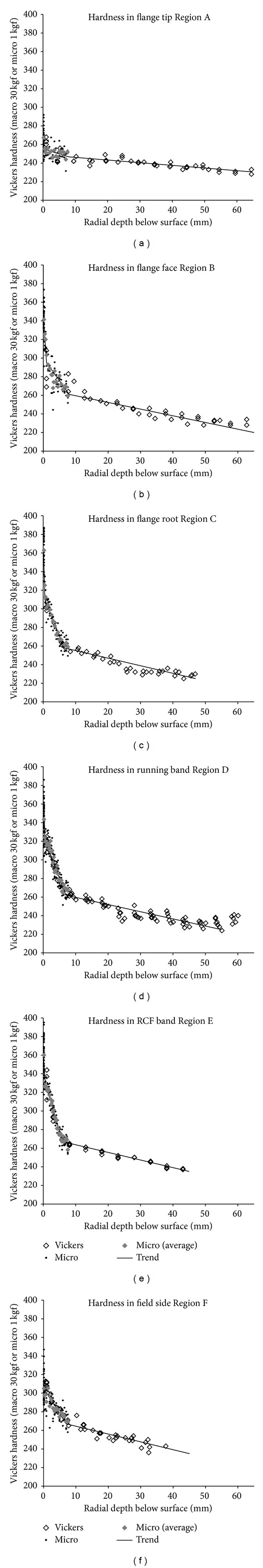
(a) Hardness trend, regional DMU, in flange tip (A). (b) Hardness trend, regional DMU, in flange face (B). (c) Hardness trend, regional DMU, in flange root (C). (d) Hardness trend, regional DMU, in running band (D). (e) Hardness trend, regional DMU, in RCF band (E). (f) Hardness trend, regional DMU, in field side (F).

The lower part of Figure 8 shows hardness values typically in the range from 230 HV to 250 HV, with a slight trend of increasing hardness toward the surface and toward the right-hand (field) side of the wheel. Nearer the running surface, the hardness increases significantly.  Figure 9 shows the hardness measurements as a function of depth for the six regions A to F across the wheel. In each region the hardness varies with depth according to a three-stage trend. However, the characteristics of these trends are different for each region of the wheel.

## 4. Observations of Microstructural Shear

**Figure 10:**
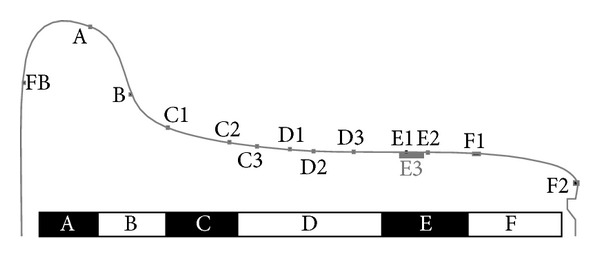
Locations of microstructural shear images on the wheel tread cross section. Note that the machined groove on the rim face indicates the minimum wheel diameter.

**Figure 11:**
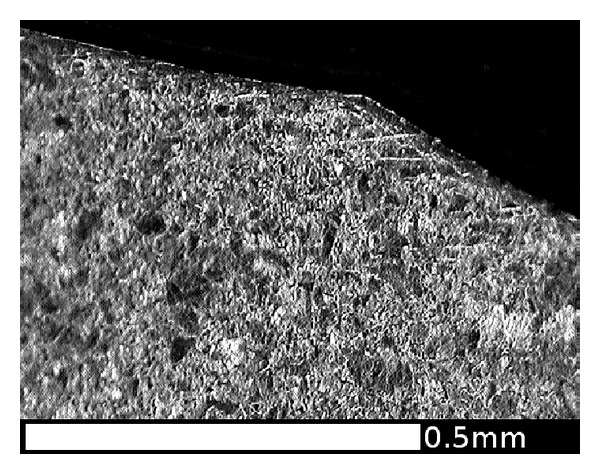
OM image of microstructure on the flange tip (A); this region does not contact the rails, and the surface shape retains the machining marks from the last turning on the lathe. There is no obvious shearing of the microstructure.

**Figure 12:**
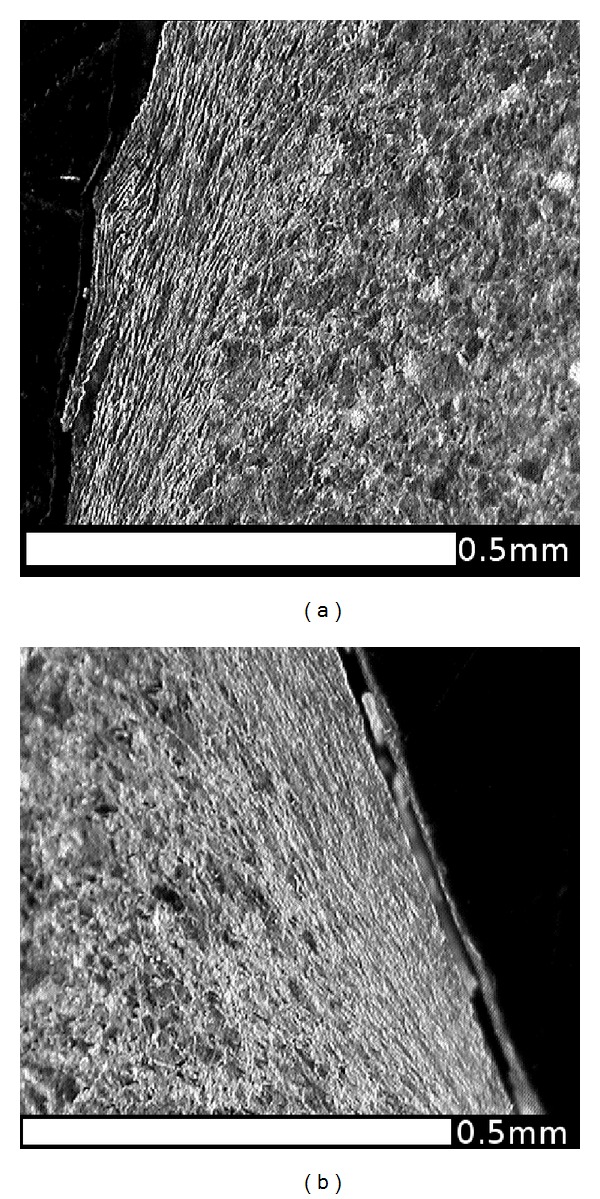
OM images of microstructure: (a) flange back (FB) and (b) flange face (B); These regions experience rubbing contacts with checkrails or the outer running rail on sharp curves. The predominant direction of the shear forces is circumferential (normal to the cross section in the figure), and the microstructure is affected to a depth of approximately 0.2 mm.

**Figure 13:**
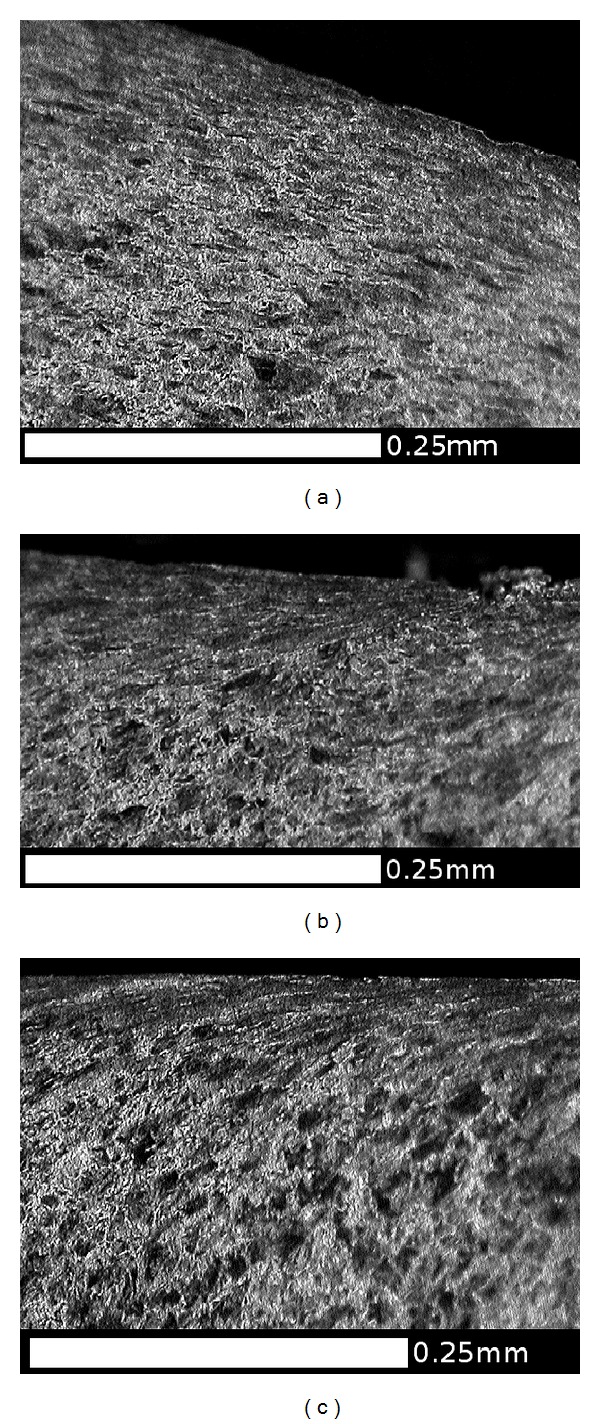
OM images of microstructure in the flange root area: (a) Region C1, (b) Region C2, and (c) Region C3. These show the microstructure sheared to the right (toward the centre of the tread) which is consistent with the direction of tangential forces on this part of the wheel (Figure 4). The depth of shearing is about 0.1 mm. A crack is visible in Region C2, at the same angle as the sheared microstructure, about 0.2 mm long. Cracks are also visible on the surface here (see Figure 3).

**Figure 14:**
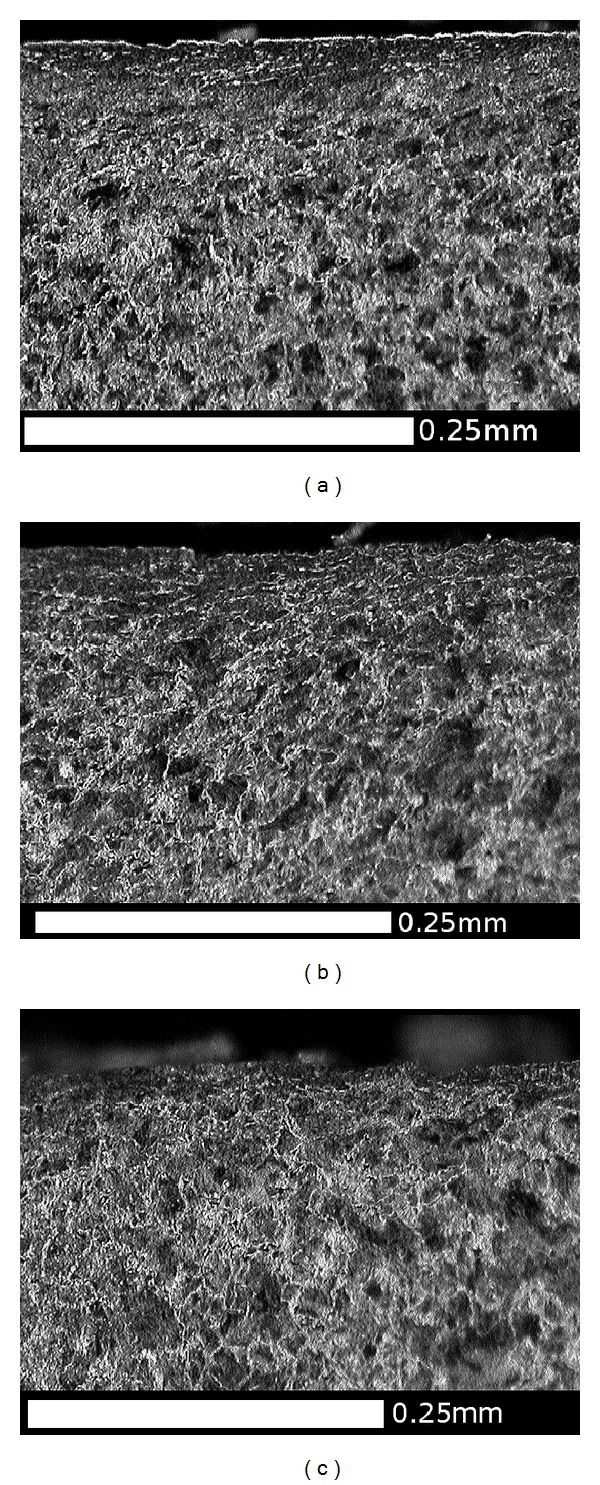
OM images of microstructure in the running band (centre of the tread): (a) Region D1, (b) Region D2, and (c) Region D3. The tangential forces are generally lower in this region and typically circumferential (normal to these sections). There is no obvious shearing of the microstructure here.

**Figure 15:**
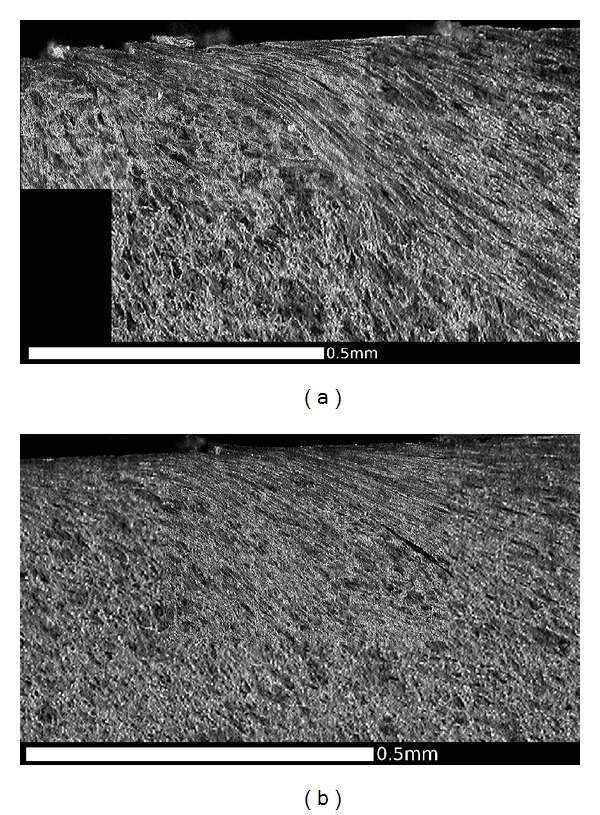
OM images of microstructure in the RCF band, (a) Region E1, and (b) Region E2. This region experiences high tangential forces, typically toward the centre of the tread (a) as shown in Figure 4, and these cause significant surface cracking (Figure 3). The microstructure is sheared in the same direction, and the influence extends to a depth of about 0.3 mm. Cracks are visible in this section, including two that have joined sub-surface in Region E1.

**Figure 16:**
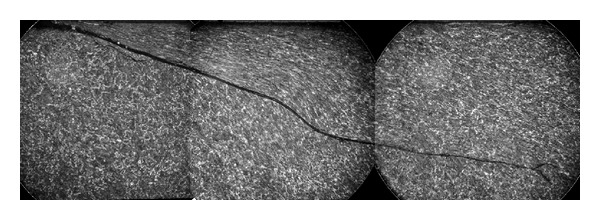
Composite OM image showing RCF crack in Region E3, viewed in a circumferential direction on a cross section. Running surface at top of image, field side to right. Crack length 6.2 mm, depth 2 mm. Note branching at crack tip and deformed microstructure above crack. This image is from a different cross section of the same wheel shown in Figure 14, selected to show a longer crack.

**Figure 17:**
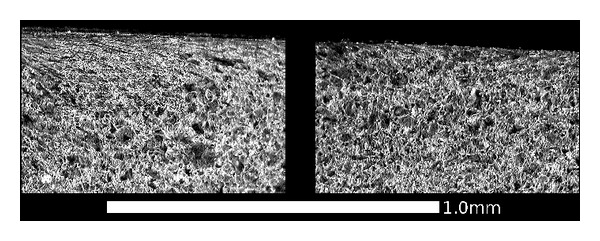
OM images of microstructure toward the field side of the tread (Region F1), showing the transition from sheared material (left, bordering the RCF band) to unsheared material in the region that is rarely in contact with the rails. The majority of Region F does not exhibit shearing of the microstructure.

**Figure 18:**
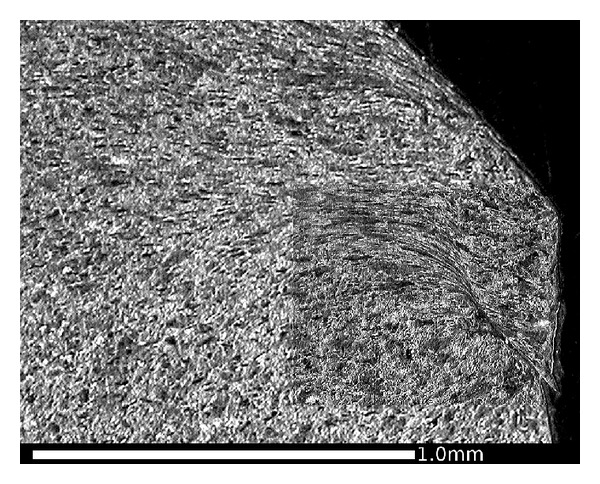
OM image of microstructure on the field side tread chamfer, showing localised plastic flow (Region F2). Together with the lack of microstructural shear in the majority of Region F, this suggests that material “rollover” on the field side of this wheel is a localised phenomenon and not associated with material flow from other parts of the wheel tread.

Another slice of the same wheel described in the previous section was cut into smaller sections, and the radial surfaces were mounted in conducting Bakelite, ground, and polished to a 1 micron finish then etched in 2% nital. The microstructures were observed using an optical microscope (OM) and in a JEOL 7000 scanning electron microscope (SEM). The regions near the running surface of the wheel tread were examined to identify the presence, direction, and depth of visible lateral shearing of the microstructure. This process also identified a number of locations where cracks had propagated, generally parallel to the sheared grain boundaries. A number of locations were identified to represent the different observations across the wheel tread cross section.  Figure 10 shows these locations in relation to the wheel tread profile; the black area at each marker represents the area shown in the corresponding microstructure image. A larger composite image marked as a grey area on Figure 10 was taken from an adjacent section which intersected a significant RCF crack. The images are shown in the subsequent Figures 11 to 18 and described in the captions; in all cases the orientation is the same as in Figure 10.

## 5. Discussion

### 5.1. Underlying Hardness Trend: Ferrite Fraction and Pearlite Lamellae

Figure 9 demonstrates that in all six regions of the wheel rim section there is a gradual decrease in hardness with depth, below 8 mm under the running surface. This is similar to that shown in Figure 2 for a new wheel section, a consequence of the wheel manufacturing process.

**Figure 19:**
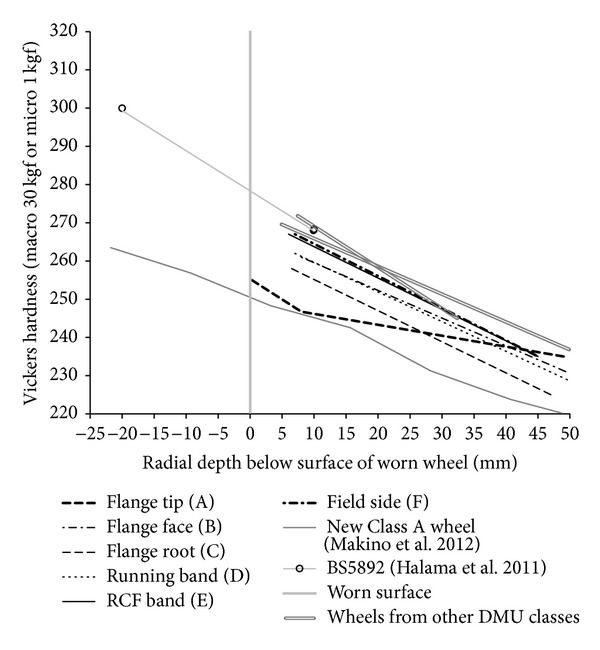
Comparison of underlying hardness trends.

The flange tip (Region A) does not experience contact forces in operation. In this region, the underlying hardness trend continues almost to the surface. A small degree of near-surface hardening is evident which may be a result of the machining process on the wheel lathe.  Figure 11 shows that there is no significant shearing of the microstructure in this region and that the machining marks are still clearly visible.  Figure 19 compares the underlying hardness trends in each region with the requirements of BS5892 [28] and the measurements from a new wheel of a slightly softer steel [19]; the new wheel surface would be at −25 mm on the *x* axis.

**Figure 20:**
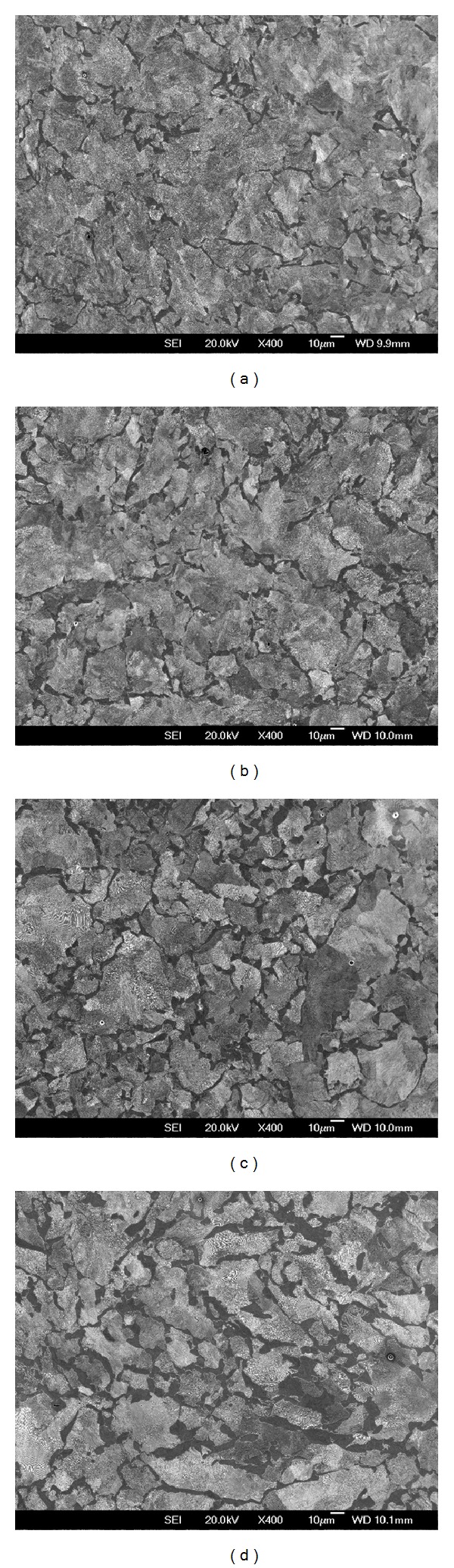
(a) SEM image of microstructure, Locn 1 on Figure 8. PE Ferrite 8.8%, 271 HV. (b) SEM image of microstructure, Locn 2 on Figure 8. PE Ferrite 9.8%, 255 HV. (c) SEM image of microstructure, Locn 3 on Figure 8. PE Ferrite 16.9%, 237 HV. (d) SEM image of microstructure, Locn 4 on Figure 8. PE Ferrite 20.9%, 232 HV.

**Figure 21:**
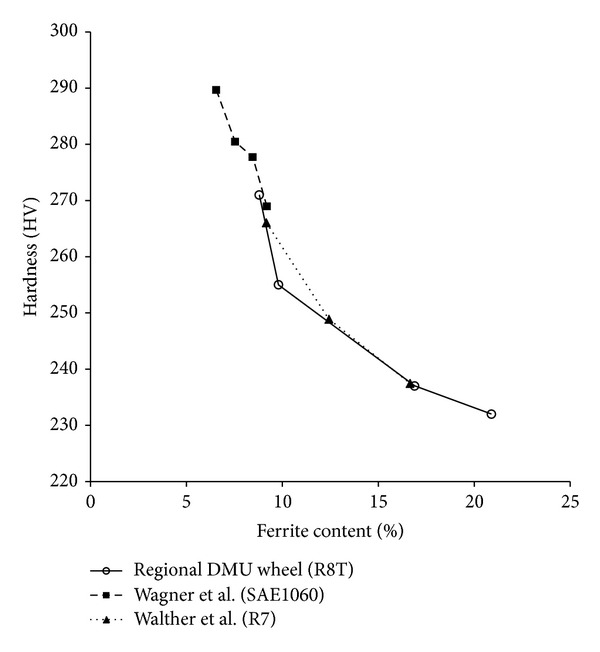
Relationship between PE ferrite fraction and hardness.

Figure 20 shows the microstructure at the locations 1 to 4 marked on Figure 8, covering the range of the underlying trend in hardness. The carbon content is usually fairly consistent through the rim [29]. The dark areas in Figure 20 represent proeutectoid (PE) ferrite, and the area fraction (equivalent to volume fraction) of PE ferrite increases with depth. Previous work [18, 32] has correlated hardness measurements in this portion of railway wheels with observed PE ferrite fraction.  Figure 21 shows how the results from the regional DMU wheel correlate well with those for similar steel grades by Wagner et al. [32] and Walther and Eifler [18].

**Figure 22:**
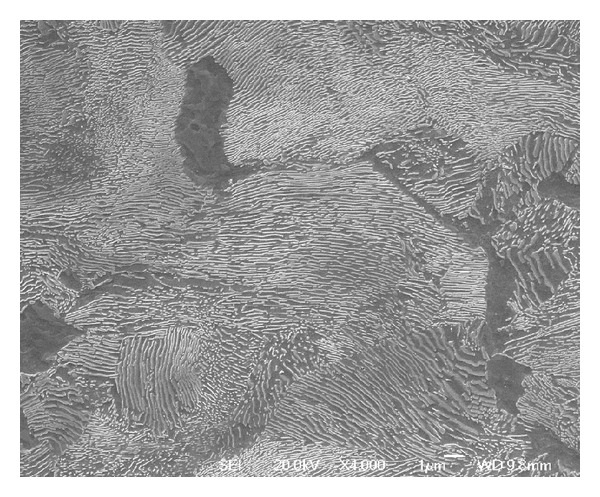
SEM image of pearlite lamellae in Region 1 of the wheel.

The local cooling rate of the material during the manufacturing process may also influence the spacing of the pearlite lamellae, with a smaller spacing corresponding to a higher hardness.  Figure 22 shows the pearlite lamellae in location 1 on Figure 8. There is some variability in the apparent spacing, but much of this is due to the oblique orientation of many grains relative to the observed surface. The smallest spacing observed is an approximation of the true value [35], and is approximately 0.10 *µ*m on Figure 22. Considerable work has been undertaken to relate the lamellar spacing to mechanical properties, for example, [36]. The relationship between lamellar spacing and hardness of this wheel sample is broadly in line with those quoted for rail steels [37].

Considering the above issues, the underlying trend in hardness is likely to be a consequence of the manufacturing process and is influenced by the local PE ferrite fraction and pearlite lamellar spacing of the material.

### 5.2. Near-Surface Characteristics

Figure 9 shows that the highest hardness values are found in the near-surface layer (generally less than 1 mm deep). This layer is work-hardened by running to a level approaching the applied stresses, and plastic flow is apparent from observations of the deformed microstructure as shown in Figures 11 to 18.

This effect is most significant in the regions of the wheel where there are frequent high tangential forces when the wheelset is in the leading position in the bogie (C and E). In these regions, the highest shear stresses are at the running surface, in a direction generally toward the centre of the wheel tread as shown in Figure 5. The average surface hardness in this region is around 360 HV, with local maxima close to 400 HV. This compares to the as-manufactured hardness in this region of approximately 260 HV.  Figure 13 shows the microstructure in Region C, which is sheared to a depth of about 0.1 mm. One of the fine surface cracks visible in this region on Figure 14 is also apparent on the cross section in Figure 13; this crack does not extend below the sheared region. Evidence from the wheel lathe suggests that the cracks in this region of the wheel are shallower than 1 mm.

Figures 15 and 16 show the deformed microstructure in Region E; again this is sheared toward the centre of the tread, but the deformation is deeper, typically at least 0.5 mm.  Figure 15(a) shows a location where two small RCF cracks have joined together about 0.3 mm below the surface; this could lead to the intervening material becoming detached and forming a small cavity.  Figure 16 shows a more significant RCF crack which has propagated to a depth of 2 mm (below the sheared region) and has started to branch at that depth. Evidence from the wheel lathe suggests that the cracks in this region of the wheel are often deeper than 2 mm and in severe cases can be greater than 6 mm deep. They often join with other cracks resulting in large cavities on the tread. It is possible that fluid entrapment contributes to the propagation of these deep cracks in Region E [1, 2]. The presence of such cracks provides crack shielding so the shear stresses are carried by the material above the crack, which consequently shears more than the material below the cracks, as can be seen in Figure 16.

The running band (Region D) has very frequent contacts but with lower tangential forces which are typically in the circumferential direction associated with traction and braking. This region is interesting because the number of stress cycles is similar in both rolling directions, and the circumferential forces are also similar in both directions. There is no significant shearing of the microstructure visible in Figure 14 (note that shearing in a circumferential direction on the flange face was visible in Figure 12). It is possible that the tangential forces associated with traction and braking are insufficient to shear the microstructure, but it is also possible that any shearing in one direction is cancelled by an equivalent opposite shearing when the wheel is rotating in the opposite direction. Twin-disc tests where the rolling direction was reversed showed this effect [7, 8]. The average surface hardness here is rather less than in Regions C and E, around 340 HV.

The peak of the stress distribution in Region D is sub-surface as shown in Figure 6(b) but generally of sufficiently low magnitude that it does not cause significant damage. Exceptionally, some individual wheels on the observed fleet of trains exhibit sub-surface transverse cracks in this region as shown in Figure 7. Following removal of the damaged material by turning, this has been found to recur on the same wheel. Because this is a rare but recurrent effect on the observed fleet and has not been observed on the opposite wheel of the same axle as an affected wheel, it is more likely to be influenced by a manufacturing or material defect in the wheel than by operating conditions or bogie setup.

The flange face (Region B) experiences less frequent contacts, often lubricated but with high creep velocity and therefore high shear forces. The average surface hardness is around 340 HV. The microstructure in Figure 12 suggests that the direction of shear is predominantly circumferential (normal to the section). The wear rate is more significant in this region.

Region F has experienced only a few thousand stress cycles, compared to the millions of cycles on other parts of the wheel tread. It does not show a significant degree of plastic flow or surface hardening.  Figure 17 shows the boundary between the region where material is sheared toward the centre of the tread and the region where there is no observable shear. A similar effect is visible in the surface hardness in Figure 8. Previous work based on twin-disc tests [3] indicates how the number of stress cycles influences the near-surface hardening and accumulated shear strain; a few thousand cycles under the conditions tested had a relatively small hardening effect. On the field-side chamfer, there is another region of high hardness in Figure 8; examining the microstructure in this region (Figure 18) confirms significant localised plastic flow.

When running, material is also removed by wear on all regions of the wheel tread; on the tested wheel this accounts for up to 2 mm loss of thickness. The highly-deformed material at the surface of the sample had been about 2 mm below the surface when the wheel was last turned on the lathe. It is hoped to consider the influence of wear in more detail in future work, perhaps using “brick model” techniques [4].

One notable observation in the surface layer of the regional DMU wheel was a region of “white etching layer,” in the centre of the running band (marked “W” on Figure 8). This was approximately 5 mm wide and 0.15 mm deep, with hardness in the range from 780 HV to 900 HV. This appears to be associated with a prolonged wheel-spin incident, which was recorded by the on-train systems during low adhesion conditions two days before the wheel was taken out of service. The hardness values from this layer have been excluded from the results presented here. This incident does not appear to have had a significant influence on the material properties in the wheel beyond the immediate vicinity of the white layer; wheels without the white layer tested by the authors show an otherwise similar pattern of hardness.

### 5.3. Hardness Gradient in the 1–7 mm Depth Range

The previous sections have identified the underlying hardness gradient as a consequence of the manufacturing process and PE ferrite content and the near-surface hardening related to the gross plastic flow caused by contact stresses in operation.

The 1–7 mm depth band between these two areas shows a very distinct hardness gradient in Figure 9, especially underlying the zones of frequent wheel/rail contacts (regions C, D, E), but is not so obviously deformed at the microstructural level.

Studies of used rails [20] have also found that the depth of hardening is greater than the depth of visual microstructural deformation. Hardness is influenced by factors that do not result in a macroscopic change in the microstructural appearance, such as the increase in dislocation density through work hardening.

**Figure 23:**
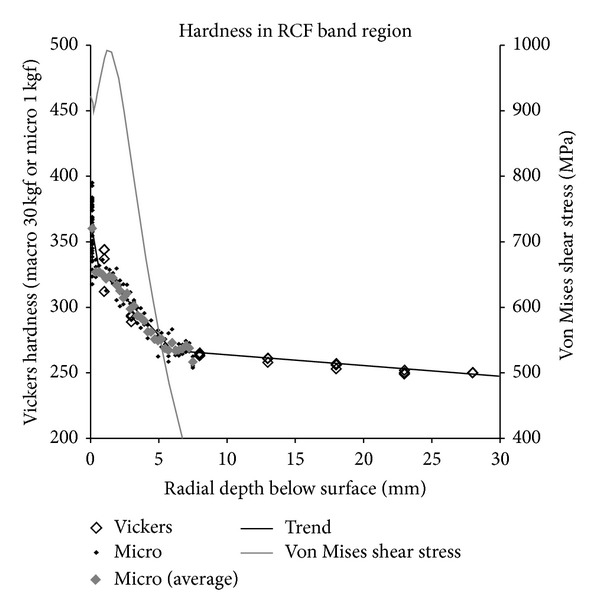
RCF band: relationship between predicted maximum von Mises shear stress and hardness in worn wheel.

Figure 23 (for the RCF band) compares the measured hardness values to the maximum von Mises shear stress at a given depth derived from the simulations (Figure 7). The Vickers hardness value of the material is approximately half the yield stress in shear [38] so the parameters have been plotted on scaled axes to give an effective comparison.

It is clear from Figure 23 that the depth of the hardening effect corresponds closely to the depth at which the applied shear stress exceeds the shear stress of the undeformed material.

Based on a comparison of the dynamic simulations and material observations in this work, it appears that the material in this layer hardens towards the applied stress level. The predicted stresses in the RCF band (Region E) are considerably higher than those in the running band, which may account for the increased hardening effect in Region E. The properties in this layer may be important as it is this material that becomes the surface when the wheelset is turned on the lathe. Also, RCF cracks in Region E can propagate down into this layer.

### 5.4. Influence of the Material Properties on RCF Crack Initiation

The susceptibility of railway wheels to RCF damage is influenced by the properties of the wheel material. On the monitored fleet, RCF damage is observed to form much more rapidly on wheels which are reaching the end of their life. One of the potential influencing factors is the change in material properties (hardness and microstructure) with depth below the as-manufactured tread surface.

The wheel surface layer is work-hardened by running to a level approaching the applied stresses, while the cyclic loading in the regions of the wheel where there are frequent high tangential forces (C and E) causes high strains and microstructural shear. Although the hardness values and stresses represent the average behaviour, the variations in microstructure and applied stresses can lead to continuing localised damage.

As discussed in Section 5.1, the proeutectoid ferrite fraction increases through the depth of the wheel, corresponding with the underlying decrease in hardness and related to the manufacturing process. Previous work on rail steels of similar composition [39] identified a link between the PE ferrite fraction and the RCF resistance of the material, which can be summarised as follows.

- PE ferrite strain hardens more than the pearlite during cyclic loading.
- An increase in the amount of PE ferrite reduces the fatigue life during twin-disc tests.
- Fatigue crack initiation primarily occurs along highly strained PE ferrite boundaries.

Consequently, it is expected that surface cracks are more likely to initiate when the wheel is towards the end of its life, after wear and turning have removed material owing to the near-surface material having a lower hardness and higher proportion of PE ferrite.

### 5.5. Comment on RCF Crack Propagation

The previous section focused on RCF crack initiation in wheels. Fine surface cracks are often seen on wheels, but if they do not propagate below the near-surface region, they do not have a significant influence on the costs and risks associated with wheel tread damage. For example, the cracks in Region C, Figures 4 and 13(b) are of no concern in practice.

In Region E, cracks can propagate to a depth of several millimetres (Figures 4 and 16). Frequent turning of wheelsets to control the growth of these deeper cracks is a significant economic problem and can have safety implications. Further work is needed to understand the mechanisms of RCF crack propagation in wheels. This is complicated by the fact that the cracks can be of similar dimension to the wheel/rail contact patch and their presence can therefore significantly influence the stress distribution in the vicinity.

## 6. Conclusions

It has been shown that the measured hardness through the depth of an “in-service” wheel rim varies significantly, with three distinct effects.

- The underlying hardness trend (hardness decrease with depth below the tread, excluding contact stress effects) can be related to the wheel manufacturing processes and microstructural changes such as the proeutectoid ferrite fraction and pearlite lamellae spacing.
- The near-surface layer (generally less than 1 mm deep) exhibits plastic flow and microstructural shear as a consequence of wheel/rail contact forces which can considerably exceed the yield stress of the undeformed material. Observations of the deformed microstructure show significant shear deformation in the regions of the wheel which experience high tangential forces when curving, with consequentially higher hardness values. These regions also feature surface-initiated cracks, and material is lost from the surface by wear. The central running band of the wheel experiences frequent contacts in both running directions with lower tangential forces; wear occurs but the microstructure does not exhibit significant deformation.
- In a layer approximately 1 mm–7 mm deep, the wheel/rail contacts cause stresses in excess of the (undeformed) material yield stress, leading to work hardening. This causes increased hardness but does not result in a macroscopic change in appearance in the microstructure. Generally, the material in this layer appears to harden to the applied stress level. Observations of cracks in wheel sections from this fleet suggest that cracks only propagate down into this layer in Region E, the field side RCF band. This is supported by analysis of wheel-turning trends on the whole fleet and other similar fleets. In this region, the relative directions of rolling and tangential force tend to open the crack before it enters the contact patch, which might promote a fluid entrapment propagation mechanism.

The change in material properties through the depth of the wheel rim (hardness, proeutectoid ferrite fraction, and lamellar spacing) would tend to increase the likelihood of rolling contact fatigue crack initiation on wheels toward the end of their life. This correlates with the observations from a number of different fleets. However, there are other factors which could contribute to this trend and will be investigated as part of the ongoing research programme.

## Figures and Tables

**Table 1:** Inputs to Vampire dynamic simulations.

Vehicle model	Based on manufacturer's data and validated against static and dynamic test results
Track geometry	68 km route measured by track recording car
Wheel profiles	9 wheel profile pairs measured from the vehicles in varying wear states (new to prereprofiling)
Rail profiles	8 measured rail profile pairs from the route, applied according to curve radius band
Wheel/rail friction	Top of rail considered dry; flange face friction reduced locally at track-side lubricator locations
Speed profile	Based on on-train data recorder information
Traction and braking forces	Based on on-train data recorder information

**Table 2:** Estimated number of stress cycles in each region (shown in Figure 3) of the wheel since last turning (*≈*170,000 miles).

Region		Leading	Trailing
A	Flange tip	0	0
B	Flange face	1 × 10^6^	1000
C	Flange root	1.5 × 10^6^	10000
D	Running band	11 × 10^6^	13 × 10^6^
E	RCF band	5 × 10^6^	2 × 10^6^
F	Field side	1500	1500
